# Supplementary figures and images for: Pseudomonas aeruginosa Can Diversify after Host Cell Invasion to Establish Multiple Intracellular Niches
Source: mBio. 2022 Nov 14;13(6):e02742-22. doi: 10.1128/mbio.02742-22 (PMC9765609; doi:10.1128/mbio.02742-22)

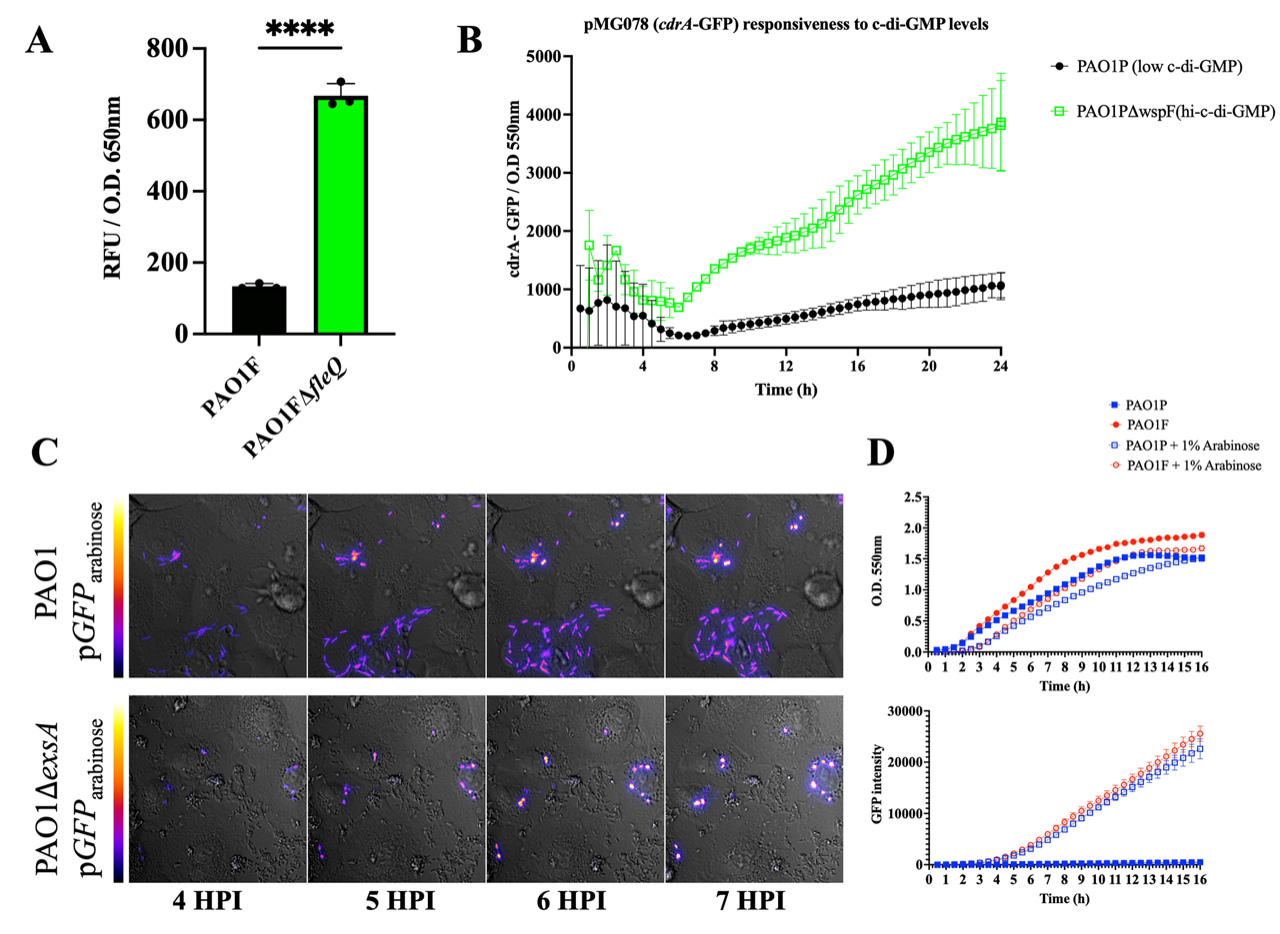

Supplement: FIG S1 [file mbio.02742-22-s0001.tif]

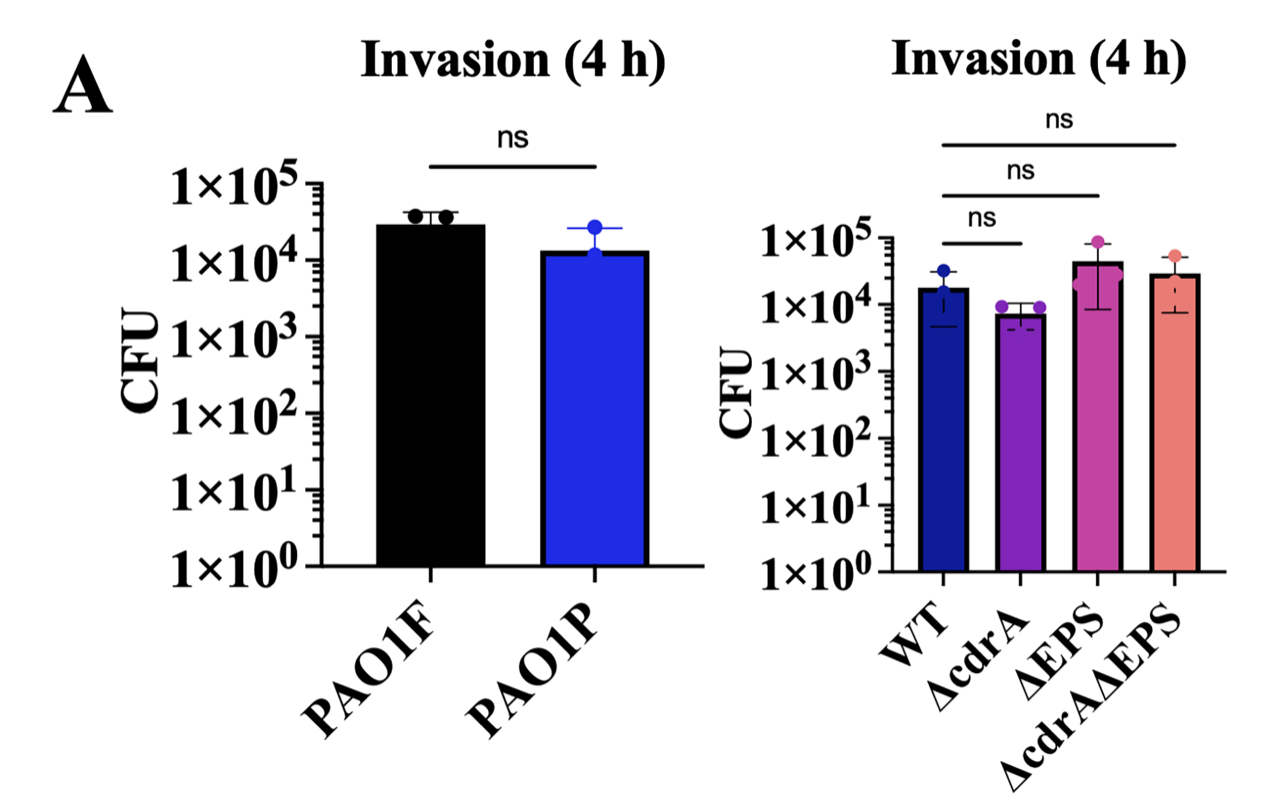

Supplement: FIG S2 [file mbio.02742-22-s0002.tif]
